# Viral Encephalopathy and Retinopathy in Dusky Groupers (*Epinephelus marginatus*, Lowe 1834) from Two Marine Protected Areas of the Northern Mediterranean Sea

**DOI:** 10.3390/vetsci13010095

**Published:** 2026-01-18

**Authors:** Enrico Volpe, Luciana Mandrioli, Riccardo Napolitano, Manuel Garcia Hartmann, Lorenzo Merotto, Albert Girons, Francesca Errani, Barbara Brunetti, Fabrizio Capoccioni, Sara Ciulli

**Affiliations:** 1 Department of Veterinary Medical Sciences, Alma Mater Studiorum University of Bologna, 47042 Cesenatico, FC, Italy; enrico.volpe2@unibo.it (E.V.); francesca.errani@uniud.it (F.E.); sara.ciulli@unibo.it (S.C.); 2 Consiglio per la Ricerca in Agricoltura e l’analisi dell’economia agraria (CREA) Centro di ricerca “Zootecnia e Acquacoltura” Via Salaria, 31, 00015 Monterotondo, RM, Italy; riccardo.napolitano@creagov.onmicrosoft.com (R.N.); fabrizio.capoccioni@crea.gov.it (F.C.); 3 Institute of Aquaculture, University of Stirling, Stirling FK9 4LA, UK; 4 MarLab, 06250 Mougins, France; manuel@zoo-vet.de; 5 Area Marina Protetta di Portofino, 16038 Santa Margherita Ligure, GE, Italy; l.merotto@portofinoamp.it; 6 ICTIOVET S.L., 08025 Barcelona, Spain; albert.girons@ictiovet.com; 7 Department of Veterinary Medical Sciences, Alma Mater Studiorum University of Bologna, 40064 Ozzano dell’Emilia, BO, Italy; b.brunetti@unibo.it

**Keywords:** grouper, *Epinephelus marginatus*, viral encephalopathy and retinopathy, nervous necrosis virus, global warming, climate change, betanodavirus

## Abstract

Betanodavirus infection poses a significant threat to marine fish species in the Mediterranean, affecting both aquaculture and wild populations. Despite increasing evidence of viral circulation in farmed and wild fish, data on natural outbreaks in wild groupers remain limited. This study investigated mortality episodes in wild dusky groupers (*Epinephelus marginatus*) within two marine protected areas (MPAs): Portofino MPA (Liguria, Italy) and Larvotto MPA (Principality of Monaco) during 2018–2019. Pathological examinations and virological diagnostics confirmed that the causative agents were betanodavirus strains belonging to the RGNNV genotype. Phylogenetic analyses revealed high genetic similarity among viral strains detected at geographically distant sites and across host species, suggesting potential regional connectivity mediated by mobile vectors or environmental transport. Seawater temperature analysis indicated that extreme and prolonged high-water temperatures were prodromal and coincided with observed outbreaks, supporting a role for thermal stress in triggering outbreak onsets. These findings highlight the susceptibility of wild dusky grouper populations to betanodavirus and underscore the interplay between host behavior, environmental conditions, and pathogen dynamics. The study emphasizes the importance of integrated health surveillance strategies within and around MPAs to monitor fish health and environmental parameters, thereby conserving wild fish populations and biodiversity.

## Simple Summary

Marine protected areas are designated regions set aside for the long-term conservation of marine life, habitats, and ecosystems. Several fish populations can benefit from the protection of these areas, including groupers, increasing their numbers and sizes. Wild dusky groupers are large predatory fish in the Mediterranean Sea, valued for their ecological role and for recreational and commercial fishing. Like other marine species, they can be affected by viral diseases that cause mass mortalities, such as viral encephalopathy and retinopathy caused by betanodaviruses. This study investigated mortality events among wild dusky groupers in two protected areas along the coasts of Italy and the Principality of Monaco in 2018–2019. Pathological and virological investigations confirmed that betanodavirus strains were responsible for the groupers’ death episodes. Closely related viruses were found in fish over 150 km apart, suggesting that the virus can spread across long distances, possibly via mobile vector species or environmental transport. Disease outbreaks coincided with periods of unusually warm seawater, indicating that rising temperatures may increase infection severity. These findings improve understanding of viral diseases in wild fish and highlight the importance of monitoring fish health and environmental conditions in marine protected areas.

## 1. Introduction

Groupers are marine predatory fish formerly classified within the family Serranidae and, more recently, placed in the family Epinephelidae, with the dusky grouper (*Epinephelus marginatus*) being the most common species in the Mediterranean. *E. marginatus* are demersal animals widely distributed in the Mediterranean Sea and adjacent Atlantic regions [1,2]. Dusky groupers are of high ecological and commercial value and are considered emblematic species of marine protected areas (MPAs) [3]. Due to their life-history traits—slow growth, long lifespan, large body size, and protogynous hermaphroditism—their populations are particularly vulnerable to overfishing and environmental stressors, and they are currently listed as endangered on the Red List of Marine Fish of the Mediterranean Sea. These fish are reef-associated species of considerable commercial and recreational relevance, sharing several biological traits that increase their vulnerability to anthropogenic exploitation [4]. In addition to direct human pressures, climate change is one of the most pervasive drivers of environmental change in marine ecosystems, with rising water temperatures emerging as a dominant factor shaping species distribution, physiology, and health [5,6]. Climate change has profoundly altered aquatic environments worldwide, affecting the abundance, spatial distribution, and phenology of several species.

In the Mediterranean Sea, sea surface temperatures have increased at approximately 0.041 °C yr^−1^ over recent decades—twice the global average—resulting in more frequent, intense, and spatially extensive marine heatwaves that profoundly affect aquatic ecosystems and their communities [7]. This warming trend is consistent with broader European patterns, where water temperatures in seas, lakes, and rivers are increasing faster than in other regions, with projected rises of 1–4 °C by the end of the century [8,9]. Temperature is one of the key environmental drivers influencing fish physiology and distribution. As ectotherms, fish maintain body temperatures equal to the ambient water temperature, making them particularly sensitive to thermal fluctuations. Predicted increases in temperature are expected to affect fish survival, growth, and population dynamics. Elevated temperatures are known to alter physiological and biochemical mechanisms, including cardiac function, oxidative stress, metabolism, and reproduction [10].

Beyond direct physiological effects, increasing water temperatures are recognized as a key factor modulating host–pathogen interactions in aquatic systems, with significant consequences for disease dynamics in both wild and farmed fish [11,12,13]. Among viral pathogens affecting marine fish, water temperature is a key driver of viral replication and disease expression in poikilothermic hosts. Elevated temperatures are frequently associated with increased incidence and severity of viral infections in both wild and farmed fish, while global warming can shift outbreak seasonality and exacerbate disease through host thermal stress and immune impairment [9,11,13].

Among viral pathogens, nervous necrosis viruses (NNVs) are of particular concern due to their broad host range and temperature-sensitive replication, which directly influences the occurrence and severity of viral nervous necrosis (VNN) outbreaks in susceptible fish species [14,15,16,17,18].

Grouper species are highly susceptible to VNN, also known as viral encephalopathy and retinopathy (VER), caused by betanodaviruses. These small, non-enveloped viruses (25–30 nm) possess an icosahedral capsid and a bipartite positive-sense RNA genome composed of RNA1, which encodes the RNA-dependent RNA polymerase, and RNA2, which encodes the capsid protein [19,20,21]. Betanodaviruses display remarkable stability across a wide pH range and environmental conditions [22,23], facilitating their persistence in marine environments. Based on RNA2 variability, they are classified into four main genotypes—SJNNV (*Betanodavirus pseudocarangis*), RGNNV (*B. epinepheli*), BFNNV (*B. verasperi*), and TPNNV (*B. takifugui*)—each adapted to distinct thermal ranges [24,25]. Reassortment between RGNNV and SJNNV genotypes is frequently observed in Mediterranean fish populations [26,27], with RGNNV displaying the broadest host range and widest distribution, including Asia, the USA, Australia, and the Mediterranean basin [22,28].

Experimental and field evidence indicates that increasing water temperatures may enhance the replication efficiency and pathogenic potential of NNV, particularly for RGNNV strains, which show optimal replication at higher temperatures (25–30 °C) [9,14,27,29]. These findings suggest that ongoing warming trends may increase the likelihood, intensity, and geographic extent of VNN outbreaks, especially in temperate regions such as the Mediterranean Sea.

To date, betanodaviruses have been detected in more than fifty fish species in the region, including European sea bass, groupers, and flatfish, with sporadic mortality episodes in wild groupers reported from various Mediterranean areas such as Italy, Spain, and Algeria [16,30,31,32,33,34,35].

In a context of rising water temperatures, open-sea farming systems typical of marine European aquaculture may become increasingly vulnerable to temperature-driven disease emergence, with potential spillover risks at the interface between aquaculture facilities and adjacent MPAs [9]. Proximity between aquaculture sites of susceptible species (e.g., European sea bass and gilthead sea bream) and surrounding aquatic ecosystems, including MPAs, raises significant concerns about potential viral transmission between wild and farmed populations [16]. Fragmentary information exists on biological factors influencing grouper susceptibility, such as weight-dependent susceptibility in orange-spotted grouper [36] and natural infections in pearl gentian grouper [37], highlighting critical knowledge gaps in VNN pathogenesis, host–pathogen interactions, and environmental persistence.

Accordingly, the present study reports mortality episodes in wild dusky groupers (*E. marginatus*) recorded in 2018 and 2019 along the Ligurian coast (Italy), within the Portofino MPA and the Larvotto MPA, belonging to the Principality of Monaco. A combination of pathological investigations and molecular diagnostics confirmed that the grouper death was associated with VER and betanodavirus infection. These findings contribute to the growing evidence that betanodaviruses are endemic to the Mediterranean basin, periodically causing disease in wild grouper populations, and suggest that ongoing seawater warming may play a role in shaping the spatial and temporal dynamics of NNV infections in natural ecosystems, underlining the importance of integrated monitoring strategies in and around MPAs, combined with health and environmental surveillance.

## 2. Materials and Methods

### 2.1. Study Area and Sampling

In the present study, we describe anomalous mortality outbreaks affecting wild dusky groupers (*Epinephelus marginatus*) in the marine protected areas (MPAs) of Larvotto (Monaco Principality) and Portofino (Italy). Moribund (showing abnormal swing behavior) and dead adult dusky groupers were reported near the coast by fishermen and divers in 2018 (Monaco Principality) and 2019 (Monaco Principality and Italy).

Along the coasts of the Larvotto MPA, at least 12 moribund/dead groupers were reported in October 2018. One animal (MC2018_561) spotted moribund but still alive close to the coast was collected and kept at the MPA facility aquarium until death (48 h); the brain, eye, kidney, spleen, heart, and liver were sampled. Moreover, the brain, kidney, spleen, and heart were sampled from one already dead grouper (MC2018_560). Similarly, at the same site, another mortality outbreak involving an undetermined number of groupers was reported in October 2019. The brain, eye, kidney, heart, liver, and feces from one recently dead grouper (MC2019_684) were sampled. All samples were sent to the Fish Product Unit in Cesenatico of the Department of Veterinary Medical Sciences (University of Bologna) for analysis.

In summer/autumn 2019 (first record on 23 August), along the coasts of the Portofino MPA (Italy), a further mortality outbreak involving several tens of groupers was reported by fishermen, divers, and MPA personnel. Divers spotted some animals with abnormal swimming close to the seabed and others stationed at the surface. Some animals presented with body ulcers and ocular opacity. Mortality affected both young (20–30 cm) and sexually mature fish. On 25 October, a 64 cm long and 4.27 kg moribund animal (sample ITA2019_685) was collected from the surface by Diving Center staff, in collaboration with MPA personnel, and shipped to the Fish Product Unit in Cesenatico of the Department of Veterinary Medical Sciences (University of Bologna). Sightings of dead/moribund groupers ceased in November.

Overall, four adult groupers were retrieved from three mortality outbreaks: two located in Larvotto MPA (2018 and 2019) and one in Portofino MPA during 2019.

### 2.2. Virological Analysis

#### 2.2.1. Virus Isolation on Cell Culture

Brain samples of specimens collected during mortality outbreaks were weighed, diluted 1:9 (wt/vol) with L-15 containing 2% FBS (Gibco, NY, USA), and homogenized before centrifuging at 2000× *g* (15 min). The supernatants were incubated with 1% *v*/*v* of antibiotic-antimycotic solution (Gibco) overnight at 4 °C. The supernatants were inoculated onto 1-day-old SSN-1 cells [17] grown in 25 cm^2^ cell culture flasks. The incubation was carried out at 25 °C, and after 1 h of adsorption, the inocula were discarded. Five ml of L-15 containing 2% FBS was added, and the flasks were incubated at 25 °C for one week. Monolayers were observed daily under a microscope for cytopathic effect (CPE) for 1 week. Cell culture supernatants with CPE were collected and stored at −80 °C.

#### 2.2.2. Molecular Investigations, Genetic Characterization, and Phylogenetic Analysis

All the tissue samples (20–30 mg) collected from the specimens were homogenized, and RNA was extracted according to the manufacturer’s instructions using the PureLink RNA Mini Kit (Invitrogen, Carlsbad, CA, USA). Tissue samples analyzed from each animal were reported in Table 1. The presence of betanodavirus was investigated by a real-time RT-PCR protocol [21]. The real-time RT-PCR assay was carried out according to Baud and colleagues [21] using the specific primers oPVP154 (5′-TCCAAGCCGGTCCTAGTCAA-3′), oPVP155 (5′-CACGAACGTKCGCATCTCGT-3′), and probe (Cy5-CGATCGATCAGCACCTSGTC-BHQ2). Briefly, the reaction mixture contained 1× Quantitect RT-PCR master mix (Qiagen, The Netherlands), 600 nM of each primer, 400 nM of the probe, and 2.5 μL of RNA in a total volume of 12.5 μL. The thermal cycling conditions were 30 min at 50 °C, followed by 15 min at 95 °C, and 40 cycles of denaturation/extension for 15 sec at 94 °C and 60 sec at 60 °C.

Brain samples that resulted positive for betanodavirus in the real-time RT-PCR were further analyzed using two RT-PCR protocols previously reported in the literature [38,39]. Viral RNA was amplified through a one-step RT-PCR assay employing either the primer pair VNNV5 (5′-GTTGAGGATTATCGCCAACG-3′) and VNNV6 (5′-ACCGGCGAACAGTATCTGAC-3′) as described by Toffolo et al. [38], or primers S6 (5′-ATGGTACGCAAAGGTGATAAGAAA-3′) and S7 (5′-GTTTTCCGAGTCAACACGGGT-3′) described by Ciulli et al. [14] targeting RNA1 and RNA2, respectively. The resulting PCR amplicons were cleaned using ExoSAP-IT^®^ (Affymetrix, Santa Clara, CA, USA) following the manufacturer’s recommendations and subsequently submitted for sequencing at the Bio-Fab Sequencing Service (Rome, Italy). Pairwise nucleotide and amino acid identities for the obtained RNA1 and RNA2 sequences were calculated with BioEdit to compare the viral strains isolated in this study and viruses detected during previous Mediterranean grouper VER outbreaks and from farmed finfish. Furthermore, sequence data obtained for RNA1 and RNA2 were aligned and compared with betanodavirus sequences previously generated from strains detected in grouper and other wild and farmed fish species collected from several Mediterranean regions (Table S1) and available in the GenBank database (www.ncbi.nlm.nih.gov accessed on 16 December 2025). Alignments were performed using ClustalW implemented in the BioEdit package version 7.7.1 (http://bioedit.software.informer.com/ accessed on 16 December 2025). Phylogenetic relationships among the strains were reconstructed using the maximum likelihood (ML) approach with the general time-reversible (GTR) substitution model [40], as implemented in MEGA 12.1 (www.megasoftware.net accessed on 16 December 2025). Node support was evaluated using 1000 bootstrap replicates, and bootstrap values ≥ 70% were considered statistically reliable.

### 2.3. Histopathology

From all animals, samples of the several organs (MC2018_560: gills, eye and brain, liver, heart, ovary, digestive tract; MC2018_561: gills, eye, liver, brain, kidney, intestine sample; ITA2019_685: gills, kidney, heart, stomach, brain, eye, ovary, spleen) were fixed in 10% buffered formalin and processed for histopathological examination. They were dehydrated through a graded ethanol-xylene series, embedded in paraffin, and 3-μm-thick sections were stained with hematoxylin-eosin (H&E) for morphological examination. Gram, Ziehl-Neelsen, and Grocott special stains were also performed to detect bacteria and fungi in histological sections.

### 2.4. Environmental Analysis

For each study area (MPA-Portofino and MPA-Larvotto), sea-surface temperature (SST) data were obtained from CMEMS (Mediterranean Sea Physics Reanalysis). The software R version 4.4.3 was used for the analysis. All grid cells within the MPAs were considered; thus, SST daily data were extracted from 1 January 2000 to 31 January 2020. For each time step, SST values were averaged over all selected grid cells to obtain a single spatially averaged SST time series per region. For the analysis, two-time windows were considered: (1) October, corresponding to the month in which grouper mortality was recorded; and (2) July–August–September–October (JASO), representing the warmest period in the Mediterranean and immediately preceding the observed mortality events.

#### 2.4.1. SST Anomalies

To quantify SST anomalies for the two-time windows, a climatological baseline was defined using data from 2000 to 2018. This 18-year period was used to compute the mean historical SST for each study site. The resulting baseline provided a robust reference against which SST values from the target years (2018 and 2019) were compared, allowing the identification of anomalies and significant deviations from the historical average. When a target year fell within the baseline period, it was excluded from the baseline calculation (e.g., for MPA-Larvotto in 2018, the baseline was computed using data from 2000 to 2017) to avoid circularity in assessing how unusual that year was. For each day, SST values from all baseline years were averaged to derive the expected “climatological” value for that date. For the October window, data were grouped by day of the month (1–31) and, for each day, the mean and standard deviation across all baseline years were calculated. For the JASO period, the same procedure was applied using the day of the year instead of the day of the month. These metrics represent the typical SST conditions and their interannual variability.

In both cases, daily anomalies were obtained by subtracting the corresponding climatological value from the observed SST for each day. Yearly mean anomalies for October and JASO were then computed by averaging daily anomalies across all days within the respective time windows. Percentile ranks were derived by comparing the target year’s mean anomaly with the distribution of mean anomalies calculated for all baseline years.

*anom* (*t*) = *SST* (*t*) − *clim* (*t*) (1)

where *anom* (t) is the anomaly at time (t), *SST* is the observed temperature, and *clim* is the climatological SST based on the baseline (2000–2018).

To assess how unusual a given year was relative to the historical range, we constructed an empirical cumulative distribution function (ECDF) from the baseline annual mean anomalies. We used it to assign a percentile rank (0–1) to the target year’s mean anomaly.

#### 2.4.2. Heat-Stress Index

To assess whether groupers in the study area may have experienced thermal stress (i.e., exposure to exceptionally warm, prolonged water temperatures), a threshold-based cumulative heat-stress index was applied. The 90th percentile of the SST baseline climatology (see the previous section) was used as a day-specific thermal threshold, allowing it to follow the seasonal cycle (higher in midsummer, lower in early autumn), consistent with Hobday et al. [41]. All calculations were restricted to the warm season from July to October (JASO).

For each JASO day t(with day of year $d_{t}$), the cumulative heat index for year y was quantified as:

cum_heat (*y*) = ∑ max [0, *SST* (*t*) − *T*_90_(*d_t_*)] (2)

where *SST* (t) is the daily *SST* and $T_{90}(d_{t})$ is the day-specific 90th-percentile threshold. This metric, expressed in °C·days, integrates both the magnitude and duration of threshold exceedances (e.g., one day 0.5 °C above the threshold contributes 0.5 °C · 1 day; ten such days contribute 5 °C · 10 days). Conceptually, it is analogous to the “Degree Heating Days/Weeks” used by Maynard et al. [42].

## 3. Results

### 3.1. Virological Analysis

#### 3.1.1. Virus Isolation on Cell Culture

Betanodavirus infection in groupers’ brains, analyzed in this study, was confirmed by cell culture isolation. All four samples induced an NNV characteristic cytopathic effect on SSN-1 cells maintained at 25 °C, marked by extensive vacuolization of the cell monolayer.

#### 3.1.2. Molecular Investigations, Genetic Characterization, and Phylogenetic Analysis

Tissue samples obtained from the four groupers showed different results in real-time RT-PCR. Viral RNA was detected in all tissue samples collected from the analyzed groupers except for kidney, liver, and feces from specimen ITA2019_685. All four brain samples were strongly positive for betanodavirus, with the highest Ct-values. On the other hand, viral presence and loads in internal organs were variable depending on the analyzed tissue and animal and generally showed lower Ct-values (Ct 26–36) compared to nervous tissues (Ct 9–16). In one case (ITA2019_685), gill and muscle tissues were also collected and analyzed, showing the presence of viral RNA even in these non-visceral organs, albeit at very low levels (Ct 33–35). Finally, for subject MC2019_684, a low (Ct 24, 48) but not negligible release of virus through feces was determined. All the obtained Ct-values were reported in Table 1. Brain samples also tested positive for betanodavirus using both RT-PCR protocols targeting the RNA1 and RNA2 genome segments. The molecular analyses yielded partial RNA1 and RNA2 sequences from the four specimens examined (MC2018_560, MC2018_561, MC2019_684, and ITA2019_685), which were subsequently deposited in the GenBank database (accession numbers PX734140—PX734147).

**Table 1:** Ct-values obtained with real-time RT-PCR analysis. Ct-values are shown as the mean of three repeats ± standard deviation.

Organ	MC2018_560	MC2018_561	MC2019_684	ITA2019_685
Brain	14.32 ± 0.27	16.04 ± 0.26	14.15 ± 0.01	9.59 ± 0.72
Eye	nd	17.39 ± 2.48	35.54 ± 0.50	30.32 ± 0.18
Kidney	28.01 ± 0.60	30.96 ± 0.29	31.55 ± 0.07	neg
Spleen	26.85 ± 0.03	36.26 ± 1.01	nd	35.97 ± 0.36
Heart	28.44 ± 0.11	36.81 ± 0.00	30.74 ± 0.37	34.54 ± 0.51
Liver	nd	32.87 ± 0.72	28.38 ± 0.00	neg
Gill	nd	nd	nd	35.51 ± 0.77
Muscle	nd	nd	nd	33.26 ± 0.49
Feces	nd	nd	24.48 ± 0.22	neg

Comparison of the viral sequences detected in the three different outbreaks revealed high nucleotide (RNA1 98.8–99.6%; RNA2 98.5–99%) and amino acid (RNA1 99.3–99.6%; RNA2 97.0–99.2%) identity, showing a high relationship of viral strains associated with grouper mortality events at the two sites located at a distance of more than 150 km and in two consecutive years (Table 2). Monegasque and Italian strains showed the highest nucleotide identity with betanodavirus sequences from *Balistes capriscus* (RNA1 99.0–99.5%; RNA2 98.2–99.6%) and *E. marginatus* (RNA1 99.0–99.5%; RNA2 98.2–99.6%) strains detected in the Calabria region, Italy, during 2024. Comparison of betanodavirus sequences obtained in this study with sequences retrieved from groupers during previous Mediterranean VER outbreaks showed the RNA1 fragment nucleotide identity between 99.2% and 99.5% and the RNA2 variable region nucleotide identity between 93.2% and 99.6%. Nucleotide identity with betanodavirus sequences retrieved from farmed fish was between 95.1% and 97.3% for the RNA1 fragment and between 90.0% and 98.5% for the RNA2 variable region.

**Figure 1:**
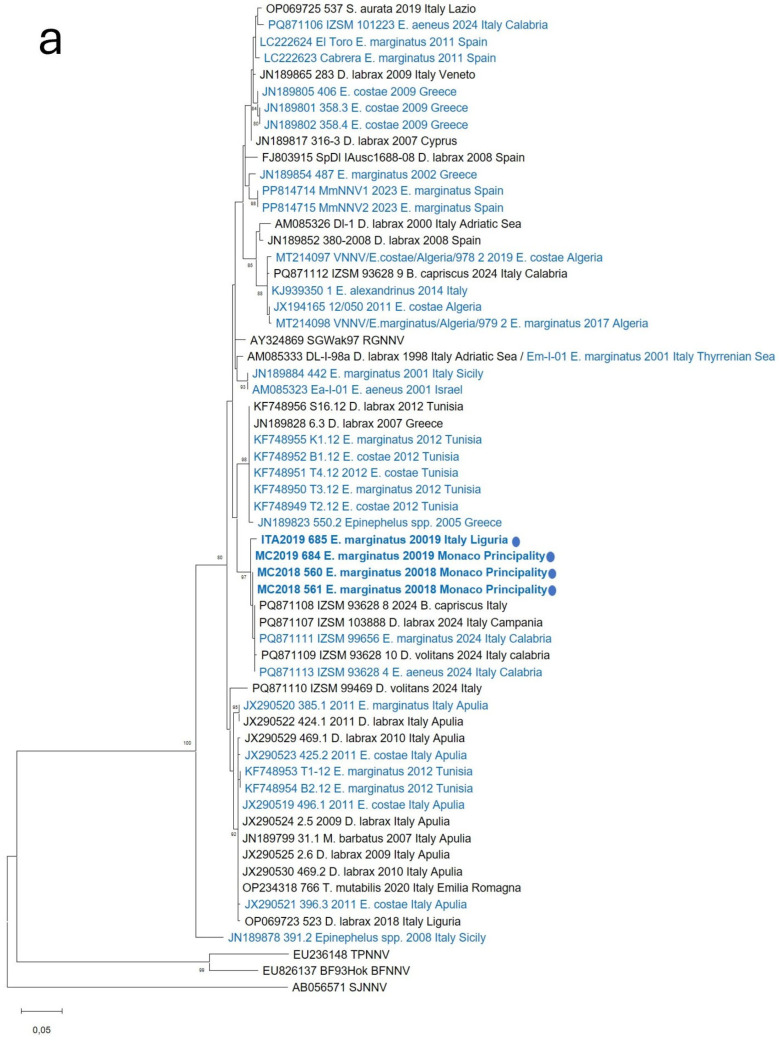

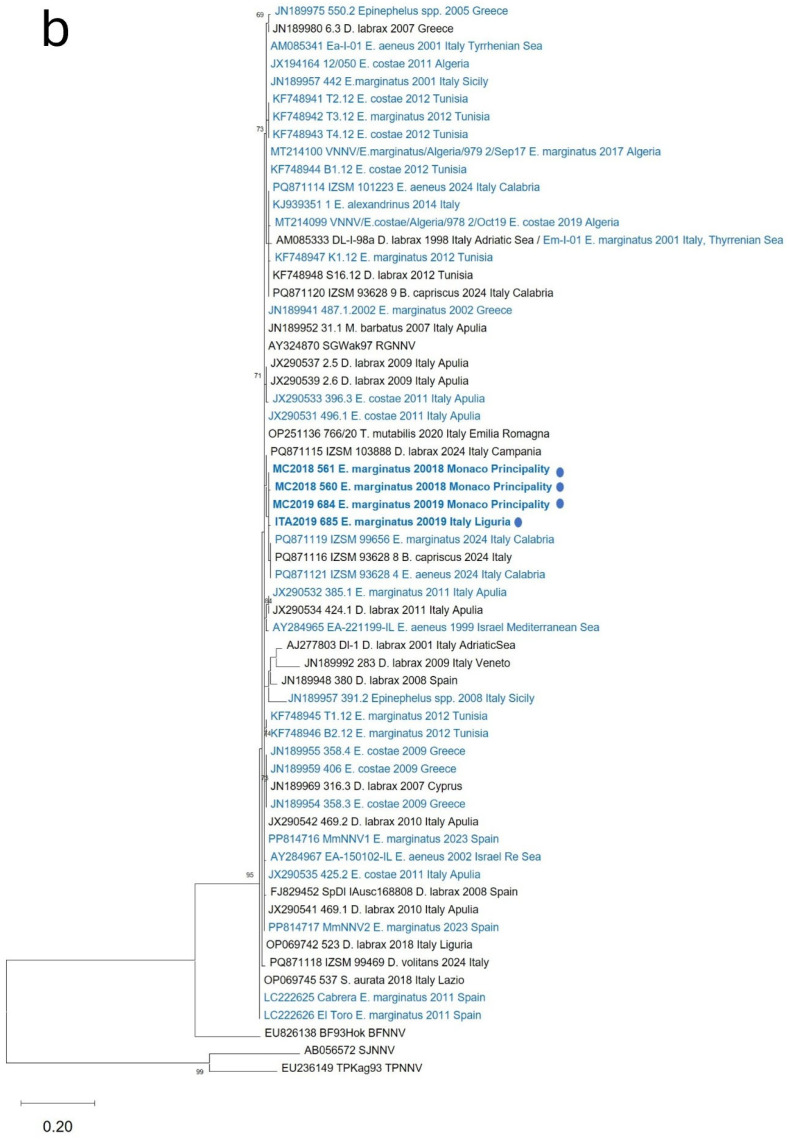
Maximum Likelihood phylogenetic trees based on RNA1 (**a**) and RNA2 (**b**) nucleotide sequences of betanodavirus strains characterized in this study from groupers (bold blue •) and selected betanodavirus sequences available on the Genbank database. Sequences retrieved from GenBank are reported with accession number, isolate name, host species, sample year, and location. Betanodavirus sequences obtained so far from Mediterranean groupers are highlighted in blue. Bootstrap values > 70% are shown. Branch lengths are scaled according to the number of nucleotide substitutions per site. The scale bar is reported.

Maximum-likelihood phylogenetic reconstruction based on the RNA1 fragment grouped the analyzed isolates into clusters corresponding to the four recognized betanodavirus genotypes. The RNA1 phylogeny positioned viruses detected in this study within the RGNNV clade (Figure 1a). Accordingly, the phylogenetic tree based on RNA2 indicated that the strains examined were grouped within the RGNNV genotype (Figure 1b). Taken together, these results suggest that viruses identified in all the samples belong to the RGNNV genotype.

Furthermore, the RNA1 phylogeny clustered RGNNV strains into subgroups that showed some geographical relationships. In particular, Italian and Monegasque strains isolated in this study clustered with recently characterized betanodaviruses detected in several wild species (*Dactylopterus volitans*, *B. capriscus*, *E. marginatus*, *E. aeneus*) in the Calabria region, Italy [43], and with a *D. labrax* strain from the Campania region, Italy. RNA2 phylogeny was less informative, with low bootstrap values, but confirmed the same clustering, showing no reassortant events in grouper betanodaviruses.

Accordingly, the RNA1 phylogeny showed that betanodaviruses previously identified in groupers also cluster with strains detected in other species, including both wild fish and farmed *D. labrax*. Similarly, RNA2 phylogeny revealed high promiscuity of grouper and other fish species betanodavirus strains, evident among all Mediterranean VER outbreaks involving groupers and showing a similar dynamic of virus circulation.

### 3.2. Gross Findings

Regarding the dusky groupers observed in the 2018 outbreak in the Monaco Principality, the subject MC2018_560 was found already dead, whereas the MC2018_561 individual was captured still alive, and a fishing hook-induced lesion was reported in the stomach.

**Figure 2:**
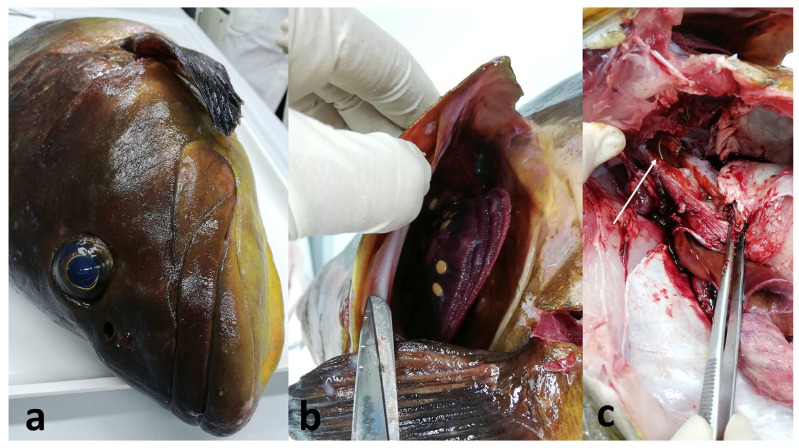
(**a**) Subject ITA2019_685 originating from the 2019 Portofino outbreak. (**b**) Gills: Trematoda parasites from the Didymozoidae family were represented by the yellow, spherical bodies. (**c**) A fishhook (white arrow) was found between the anterior portion of the celomatic cavity and the retro-opercular branchial spaces.

For subject ITA2019_685 (Figure 2), originating from the 2019 Portofino outbreak, the main findings were bilateral corneal opacity, reddened urogenital pore, some branchial parasites (belonging to the *Didymodiclinus* species) attached to all arches, swimbladder hyperinflation, and chronic peritonitis characterized by fibrous adhesions among viscera. A fishhook was found, free, at the limit between the branchial spaces and the anterior part of the celomatic cavity, without evident contact with the viscera. The stomach and intestine did not contain food; the gastric mucosa had a cerebroid appearance but was intact, whereas the muscular wall appeared thickened.

### 3.3. Histological Findings

The brain sections of subject MC2018_560 presented with mild to moderate diffuse congestion of blood vessels, as well as occasional intraparenchymal foci of necrosis. No infectious agents or parasites were detected using routine staining techniques. Regarding the internal organs, the liver showed mild hydropic hepatocyte degeneration and mild focal lymphocytic inflammation around biliary ducts.

**Figure 3:**
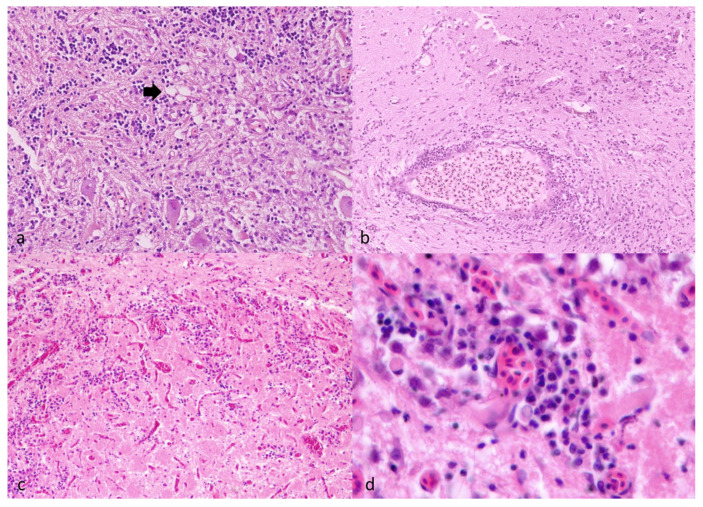
Hematoxylin-eosin staining of subject MC2018_561. (**a**,**b**) and subject ITA2019_685 (**c**,**d**). (**a**) Brain tissue showed multifocal lymphocytic inflammation of the neuropil and occasional foci of vacuolized cells (black arrow), magnification ×30. (**b**) A large vessel filled with erythrocytes (hyperemia) surrounded by small basophilic inflammatory cells consistent with lymphocytes, suggesting a virus-induced encephalitis, magnification ×40. (**c**,**d**) Glial cells were mixed with the inflammatory cells, an expression of a cerebral tissue adaptation known as juxtavascular glia hyperplasia (3c magnification ×100; 3d magnification ×400).

Brain of subject MC2018_561 presented with multifocal lymphocytic inflammation associated with meninges and congested intraparenchymal blood vessels, plus occasional focal presence of vacuolized cells (presumably neurons and glial cells) (Figure 3a). Neuropil artifacts, consistent with small cracks and increased intercellular spaces resulting from delayed fixation, were present (Figure 3).

Regarding the internal organs, the liver showed mild to moderate hydropic degeneration of hepatocytes, moderate multifocal lymphocytic inflammation of the bile ducts, and hyperplasia of melanomacrophages.

About subject ITA2019_685, the brain sections available were characterized by the presence of capillary vessels lumina filled with erythrocytes (hyperemia) (Figure 3b) and perivascular, multifocal inflammatory cells, mainly represented by lymphocytes; in addition, there was juxtavascular glia hyperplasia (Figure 3c,d), which as a whole was indicative of an encephalitis (viral-induced in consideration of the perivascular lymphocytes).

Regarding the findings in the other organs of ITA2019_685, the branchial filaments showed hyperplasia due to the presence of parasites (*Didymodiclinus* sp. and microscopic ones, consistent with *Dactylogyrus* sp. and *Gyrodactylus* sp.). Multifocal (early stage) granulomas were detected in the kidney, perivisceral adipose tissue, myocardium, and ovary.

Coccoid spherical bacteria-like organisms within the tissues, including brain capillaries, were not detected. Gram and Ziehl-Neelsen did not show the presence of specific bacteria.

### 3.4. SST Anomalies

**Figure 4:**
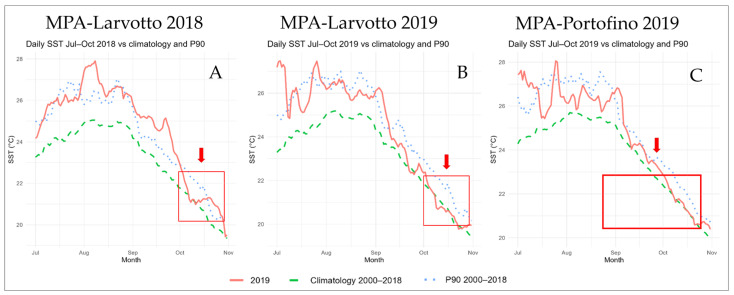
Orange lines represent the SST observed value in the target year (2019 for MPA-Portofino and 2018 and 2019 for MPA-Larvotto); Green-dashed lines represent the climatological_(2000–2018)_ SST mean of the baseline 2000–2018; Blue-dotted lines represent the 90th percentile of the climatological_(2000–2018)_ SST distribution. Red squares and arrows indicate the periods when dead events have been observed.

The results for potential SST anomalies showed distinct patterns across the two-time windows considered (October and JASO) (Figure 4). In the first case, outcomes were heterogeneous: in October 2019, at both the Portofino and Larvotto MPAs, median daily SST was close to baseline climatological conditions (+0.3 °C and +0.2 °C, respectively). In contrast, in October 2018, the Larvotto MPA displayed a marked SST increase (+0.7 °C; 90th percentile). When the four-month period (JASO) was compared with its corresponding baseline, all results were significantly higher. At Portofino and Larvotto MPAs, JASO 2019 exhibited anomalies of +0.9 °C and +1.1 °C in median daily SST (90th and 95th percentiles, respectively). During JASO 2018, Larvotto MPA also recorded the most intense anomaly, with a median daily increase of +1.53 °C (100th percentile, the warmest season on record) (Table 3).

### 3.5. Heat-Stress Index

**Figure 5:**
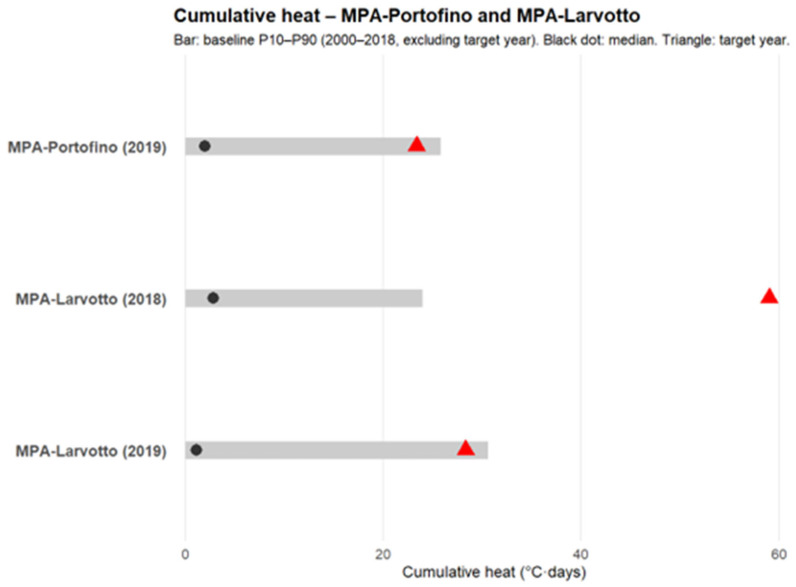
Cumulative heat index distribution based on the climatology in each site. The gray bar represents the range between the 10th and 90th percentiles for each site and year. The black dot represents the median. The red triangle represents the target year’s value.

The 90th percentile threshold–based cumulative heat-stress index, calculated for the JASO periods at the Larvotto and Portofino MPAs (2000–2019), shows that 2018 had the highest values (37.33 and 36.2, respectively). In 2019, although the index remained elevated (28.33 and 23.4, respectively), these values corresponded to the 80th percentile (Figure 5; Table 4).

## 4. Discussion

Betanodavirus infection has long been recognized as a major threat for Mediterranean marine species, affecting both aquaculture and wild populations. Since the early 1990s, recurrent episodes of viral encephalopathy and retinopathy (VER) have been reported in several countries bordering the Mediterranean basin, with increasing evidence of viral circulation not only in farmed fish but also in a wide range of wild hosts [44]. In the last decade, countries bordering the southern coasts of the Mediterranean Sea and the southern regions of Italy and Spain experienced several grouper mortality outbreaks due to betanodavirus infection [16,30,31,32,33,34]. On the other hand, no similar events have been reported so far in the Northern Mediterranean Sea. The results of the present study expand the geographical area affected by this plague, showing not only the widespread betanodavirus infection in groupers but also the high susceptibility of dusky grouper to the virus, causing severe mortality outbreaks under natural conditions. Although previous publications also report the involvement of other finfish, groupers are the species most affected by VER outbreaks in the wild [16,43], suggesting their higher species-specific susceptibility. However, their most frequent involvement in VER outbreaks could also be due to their habitat behavior, such as cave dwelling, exposing them to contaminated environments.

The integrated approach applied in this study—combining an epidemiological investigation of outbreak dynamics with an environmental analysis based on high-resolution water temperature data—provides additional insights into the role of high and prolonged water temperatures. This combined analysis helps clarify how environmental conditions may trigger VER outbreaks.

In line with previous observations from wild grouper mortality events [16,30,31,32,33,34], the animals analyzed in this study displayed clinical, pathological, and virological features consistent with VER. All examined brain samples showed high viral loads, consistent with the neurotropism of betanodaviruses, and virus isolation produced the typical cytopathic effect characterized by extensive vacuolization of SSN-1 cell monolayers. The diffuse presence of the virus across several organs, despite low loads, combined with high viral loads in nervous tissue, suggests a clinical stage of the infection. Histological examination further confirmed the presence of degenerative and inflammatory changes in the central nervous system, including perivascular cuffing, neuroinflammation, and microglial activation—findings highly compatible with the encephalitis described in previous Mediterranean grouper outbreaks. In our cases, the vacuolation of the brain, consistent with a degenerative cell change, was not the main histopathological finding, whereas the main signs were represented by hyperemia and inflammation, also reported by other authors in dusky grouper VER infection [33]. Indeed, our cases were characterized by juxtavascular microglial activation, a small population of microglia overlaying the vasculature, a position that would facilitate an immediate response to vascular and blood-borne signaling [45]. In addition, we are going to further investigate this microglia population.

Despite no exact data being retrieved on the consistency of dead animals, in all events, some tens of fish were involved. As in previous reports [16,31,32], sightings are often the result of individuals’ initiative; even if surveillance is one of the most important criteria to establish marine-protected areas no systematic targeted surveillance programs are always put in place to monitor disease incidence in wild fish such as grouper. However, increasing attention is being dedicated to wild fish health, particularly within marine protected areas, where grouper mortality outbreaks have already been reported [32,34,35].

Molecular analyses revealed that all four viruses identified in the present study belong to the RGNNV genotype, the most widespread in the Mediterranean Sea and commonly detected in both aquaculture and wild stocks. This result is consistent with the phylogenetic patterns observed in Tunisia [30], Algeria [31,33], Italy [16], and Spain [32,34], where RGNNV strains have repeatedly been identified in infected groupers. The high sequence identity among NNV strains detected in different host species, both farmed and wild, within the same geographic region indicates that closely related viral variants are co-circulating across multiple fish populations. This pattern suggests that shared regional viral lineages are maintained through interactions among diverse hosts, including asymptomatic wild species.

The detection of NNV infections in both farmed and wild fish across the Mediterranean has progressively revealed a complex epidemiological interface in which viral transmission may occur in both directions: from wild to farmed fish and from farmed stocks to surrounding wild fish populations. Early evidence of natural spillover was provided by Berzak et al. [46], who showed that wild species carried RGNNV strains genetically similar to those detected in newly stocked gilthead seabream, suggesting that wild-to-farmed transmission can occur even in offshore sites with minimal anthropogenic influence. Conversely, subsequent studies have documented the opposite process: Zrnčić et al. [47] reported that thicklip grey mullet captured near infected European seabass cages harbored viral strains nearly identical to those circulating in farmed fish, providing the first clear molecular indication of farmed-to-wild transmission. Together, these findings underscore that NNV transmission dynamics are not unidirectional but rather reflect a shared epidemiological scenario influenced by several factors (e.g., spatial proximity, hydrological connectivity, and the movement of wild species that may act as reservoirs or mechanical carriers).

In this context, the genetic similarity between the RGNNV strains detected in the Ligurian Sea and those associated with the mortality event in the Principality of Monaco—despite the >150 km distance between the two areas—suggests that viral connectivity may extend across spatial scales far exceeding the natural territorial range of dusky groupers.

Accordingly, phylogenetic analysis showed that the grouper strains isolated in this study clustered with betanodaviruses most recently characterized in new wild species, such as *D. volitans* and *B. capriscus*, in Southern Italy, at a distance of several hundred kilometers [43].

Given that *E. marginatus* is a territorial species with limited adult dispersal capabilities, long-distance viral flow is highly unlikely to be mediated by grouper movements. This strengthens the hypothesis that biological vectors could contribute to the regional-scale spread of viral strains. Species known to frequent both natural rocky reefs and aquaculture structures—such as mullets, bogues, or other schooling coastal fishes—may represent putative candidates, especially given previous reports of asymptomatic carriers capable of transmitting infection under experimental conditions.

Several studies have already suggested that wild fish aggregating around cages may act as transient reservoirs or mechanical vectors [32] and that anthropogenic structures can alter contact networks and locally amplify viral persistence in the water column. Our findings align with this scenario: even in areas without direct aquaculture influence, we observed highly similar viral strains circulating across distant locations. This pattern suggests that a broader regional connectivity exists, likely sustained by mobile marine species or by environmental transport processes. In this regard, the detection of NNV in bivalve molluscs—such as mussels collected in sites far from farms rearing susceptible finfish—further supports the idea that viral particles can be transported or accumulated well beyond zones of active outbreaks. Although mussels are not considered biological hosts of NNV, their capacity to filter, retain, and release viable viral particles suggests that they may act as passive environmental sentinels, reflecting broader patterns of viral circulation even in areas lacking direct aquaculture links [48]. Such a scenario is consistent with the persistent, low-level viral circulation observed in multiple Mediterranean regions. It highlights the importance of considering both ecological behavior and ecosystem dynamics when interpreting VER outbreaks in wild populations.

Altogether, these elements reinforce the need for integrated long-term monitoring of wild fish communities, particularly in endemic regions where baseline viral circulation appears to be stable. However, wide virus circulation and the fragility of the affected ecosystem pose a challenge in limiting VER damage. Understanding which species act as effective viral carriers, how far they move, and under which environmental conditions transmission becomes more likely will be essential to understanding the broader-scale dynamics of betanodavirus in the Mediterranean Sea [30,32,49]. Only through this robust scientific knowledge will it be possible to implement efficient management strategies essential for safeguarding the valuable wild fish stocks inhabiting marine protected areas.

Kersting et al. [34] documented episodes of mass mortality in dusky groupers and moray eels along the coast of the Columbretes Islands MPA in Spain, suggesting environmental stressors as potential drivers of disease emergence in wild populations. Similarly, Boukedjouta et al. [33], describing confirmed cases of VER in wild groupers in Algerian waters, reinforce concerns about interspecific and transboundary viral circulation.

Environmental conditions, particularly thermal stress, appear to be critical determinants of the onset of disease outbreaks in farmed and wild fish populations [50].

Temperature-induced stress has been shown to facilitate viral replication and exacerbate clinical manifestations in several marine hosts, including species of the genus Epinephelus [51,52]. In the present study, both 2018 and 2019 were characterized by pronounced sea surface temperature (SST) anomalies and high cumulative heat index values. Importantly, the cumulative heat index integrates both the intensity and the duration of thermal anomalies, thus capturing prolonged exposure to elevated temperatures rather than isolated short-term peaks.

These observations align with previous findings associating NNV-related mortality with unusually warm summers [16,34]. In particular, 2018 represented the warmest JASO window on record in the analyzed MPAs, with extreme and persistent high-water temperatures affecting the entire summer period prodromic to the observed mortality events, suggesting that the onset of VER outbreaks may be triggered not merely by short-term high temperatures but by extended periods of thermal stress, which could have lowered the resistance of groupers and increased viral replication, facilitating the occurrence of VER outbreaks.

Although sea surface temperature (SST) represents an indirect proxy for the thermal conditions experienced by adult dusky grouper (i.e., individuals typically inhabit depths of 10–50 m and may occasionally occur deeper [53]), subsurface temperatures within this depth range are generally influenced by surface forcing and tend to follow surface thermal events through the seasonal deepening of the mixed layer, with a relatively short temporal lag [54]. For this reason, SST was used as a conservative indicator of anomalous warming in this study. While depth-resolved temperature fields are available from the CMEMS Mediterranean Sea Physics Reanalysis, these data are entirely derived from three-dimensional physical–numerical models (NEMOMED12, ~4 km horizontal resolution) and do not correspond to direct in situ observations. Relying on such modeled subsurface temperatures would therefore introduce an additional layer of uncertainty related to model parameterization and spatial resolution, potentially obscuring the actual thermal conditions experienced by dusky grouper at depth.

Accordingly, we chose not to explicitly analyze temperature anomalies at depth and instead focused on SST anomalies relative to the 2000–2018 climatological baseline. Our objective was not to establish a definitive statistical or causal relationship between anomalous warming, VNN infections, and grouper mortality, also considering the limited sample size, but rather to highlight the occurrence of pronounced surface thermal anomalies during periods of exceptional mortality. Establishing a robust link between thermal stress, VNN infections, and dusky grouper mortality will require larger sample sizes, the inclusion of additional sites, and the availability of direct temperature measurements across multiple depths within the species’ bathymetric range. Notably, the affected individuals were mainly adults, which are generally considered more resistant to NNV infection than juvenile stages, further supporting the hypothesis that disease emergence resulted from prolonged physiological distress rather than acute exposure alone. Consistent with this interpretation, the analyzed groupers displayed signs of protracted suffering, including behavioral alterations, suggesting a chronic stress condition preceding the development of VER outbreaks.

Water temperature plays a critical role in shaping the epidemiology of NNV infections by modulating viral replication kinetics, viral loads in infected tissues, and ultimately clinical severity and mortality. Controlled experimental studies indicate that RGNNV strains replicate more efficiently at higher temperatures, typically 23–30 °C, resulting in elevated viral loads, pronounced neurological signs, and increased mortality in susceptible species. Field observations align with this temperature-dependent pattern. In Croatian seabass farms, fry carrying the virus remained asymptomatic when water temperatures were below 18–20 °C, despite testing positive by rRT-PCR; however, as temperatures rose above 22–23 °C during summer, typical VNN signs rapidly emerged and mortality spiked, accompanied by drastic reductions in Ct-values indicative of higher viral loads [47]. Seasonal fluctuations in viral detection further support a temperature-driven pattern: infected fish showed high viral loads during warm months and markedly reduced loads during winter, reflecting a low-replication state at lower temperatures. Together, these findings confirm that temperature is a key environmental trigger of VNN outbreaks, influencing not only the likelihood of clinical disease but also the persistence of subclinical infections and the potential for viral transmission in aquaculture environments.

## 5. Conclusions

Overall, the findings of the present work contribute to an increasing number of reports indicating that the Mediterranean ecosystem hosts endemic RGNNV strains circulating in multiple species and occasionally causing severe mortality episodes in vulnerable wild hosts such as the dusky grouper. The combination of environmental stress, habitat overlap with aquaculture, and the ecology of long-lived territorial predators may render groupers particularly exposed to disease emergence. Strengthening coordinated monitoring programs, integrating long-term environmental data with targeted surveillance of both wild and farmed fish, and improving knowledge on the ecology of NNV reservoirs will be essential for mitigating future outbreaks and protecting emblematic species inhabiting Mediterranean coastal ecosystems.

## Figures and Tables

**Table 2:** Pairwise nucleotide (white cells) and amino acid (gray cells) identities for RNA1 and RNA2 genome fragments of viral sequences characterized in this study.

**RNA1**	**MC2018_560**	**MC2018_561**	**MC2019_684**	**ITA2019_685**
MC2018_560	ID	1	99.6	98.8
MC2018_561	1	ID	99.6	98.8
MC2019_684	99.6	99.6	ID	98.9
ITA2019_685	99.6	99.6	99.3	ID
**RNA2**	**MC2018_560**	**MC2018_561**	**MC2019_684**	**ITA2019_685**
MC2018_560	ID	99.7	98.7	98.5
MC2018_561	99.2	ID	99.0	98.7
MC2019_684	97.7	98.5	ID	99.7
ITA2019_685	97.0	97.7	99.2	ID

**Table 3:** SITE: site identifier (e.g., MPA-Portofino, MPA-Larvotto); Year: target year; Period: October (monthly analysis) or J-A-S-O (July–October time window); Climatological mean 2000–2018 (°C): baseline mean SST for the corresponding period (for the 2018 target year the baseline years were 2000–2017); Baseline SD (°C): interannual standard deviation of baseline mean SST; Target mean (°C): mean SST for the target month or period; Anomaly (°C): SST anomaly (Target mean—Climatological mean); Percentile (0–1): percentile rank of the target year’s mean anomaly relative to baseline.

SITE	Year	Period	Climatological Mean 2000–2018 (°C)	Baseline SD (°C)	Target Mean (°C)	Anomaly (°C)	Percentile (0–1)
MPA-Portofino	2019	October	21.1	0.6	21.2	0.3	0.58
		J-A-S-O	23.8	0.6	24.7	0.9	0.90
MPA-Larvotto	2018	October	20.5	0.7	21.2	0.7	0.90
		J-A-S-O	23.1	0.5	24.6	1.5	1
	2019	October	20.5	0.7	20.7	0.16	0.63
		J-A-S-O	23.2	0.6	24.2	1.1	0.95

**Table 4:** Cumulative Heat Index (CHI): represents the value of the index. Color scale from red to green (with red representing higher values and green lower ones). Percentile rank: represents the percentile value of each year according to the index distribution (highlighted in red the percentiles ≥ 90th). Rows in bold (2018 and 2019) refer to the two target years of the present study.

	MPA-Larvotto		MPA-Portofino	
Year	Cumulative Heat Index (CHI)	Percentile Rank	Cumulative Heat Index (CHI)	Percentile Rank
2000	0.10	0.42	0.1	0.4
2001	0.00	0.32	0.0	0.4
2002	0.00	0.32	0.0	0.4
2003	35.25	0.95	34.3	0.9
2004	0.07	0.37	0.0	0.4
2005	0.00	0.32	0.2	0.5
2006	10.99	0.79	12.4	0.8
2007	0.00	0.32	0.0	0.4
2008	0.56	0.47	2.0	0.5
2009	9.76	0.74	8.1	0.7
2010	4.99	0.68	6.1	0.7
2011	1.08	0.53	0.0	0.4
2012	2.80	0.58	2.8	0.6
2013	0.00	0.32	0.0	0.4
2014	29.45	0.89	20.6	0.8
2015	17.13	0.84	23.7	0.9
2016	4.85	0.63	3.0	0.6
2017	0.00	0.32	0.0	0.4
**2018**	**37.33**	**1.00**	**36.2**	**1.0**
**2019**	**28.33**	**0.8**	**23.4**	**0.8**

## Data Availability

The original contributions presented in this study are included in the article/supplementary material. Further inquiries can be directed to the corresponding author(s).

## Supplementary Materials

The following supporting information can be downloaded at: https://www.mdpi.com/article/10.3390/vetsci13010095/s1, Table S1: details of viral strains used for phylogenetic analyses [15,16,28,30,31,32,33,34,38,43,55,56,57,58,59,60].
