# Supplementary material for: Viral Encephalopathy and Retinopathy in Dusky Groupers (Epinephelus marginatus, Lowe 1834) from Two Marine Protected Areas of the Northern Mediterranean Sea
Source: Vet Sci. 2026 Jan 18;13(1):95. doi: 10.3390/vetsci13010095 (PMC12846420; doi:10.3390/vetsci13010095)
Supplement: Supplementary file 1 [file vetsci-13-00095-s001.zip › vetsci-4082721-supplementary.pdf]

Table S1. Details of viral strains used for phylogenetic analysis. The list reports the details of the betanodavirus sequences of strains isolated from groupers and other farmed and wild species in Mediterranean Sea along with representative viral strains available in GenBank.

Name	Year	Species	Farmed/ wild	Site, Country	Accession number		Reference
					RNA1	RNA2	
SJNag93	1993	*Pseudocaranx dentex*	na	Nagasaki, Japan	AB056571	AB056572	[55]
Dl-1	2000	*D. labrax*	farmed	South-Western Adriatic Sea, Italy	AM085326	AJ277803	[38]
Dl-I-98a	1998	*D. labrax*	na	North-Western Adriatic Sea, Italy	AM085311	AM085333	[38]
Em-I-01	2001	*E. marginatus*	wild	Central Thyrrenian Sea, Italy	Same sequence as Dl-I-98a	Same sequence as Dl-I-98a	[38]
Ea-I-01	2001	*E. aeneus*	wild	Tyrrhenian Sea, Italy	AM085323	AM085341	[38]
EA-221199-IL	1999	*E. aeneus*	wild	Mediterranean Sea, Israel	na	AY284965	[56]
EA-150102	2002	*E. aeneus*	farmed	Re Sea, Israel	na	AY284967	[56]
SGWak97 RGNNV	1997	*E. septemfasciatus*	na	Japan	AY324869	AY324870	[57]
TPKag93 TPNNV	1993	*Takifugu rubripes*	na	Kagawa, Japan	EU236148	EU236149	[58]
BF93Hok BFNNV	*1993*	*Verasper moseri*	na	Hokkaido, Japan	EU826137	EU826138	[58]
SpDl-IAusc1688.08	2008	*D. labrax*	farmed	Spain	FJ803915	FJ829452	[15]
31.1	2007	*Mullus barbatus*	wild	Bari, Apulia, Italy	JN189799	JN189952	[28]
358.3	2009	*E. costae*	wild	Greece	JN189801	JN189954	[28]
358.4	2009	*E. costae*	wild	Greece	JN189802	JN189955	[28]
406	2009	*E. costae*	wild	Greece	JN189805	JN189959	[28]
316.3	2007	*D. labrax*	farmed	Cyprus	JN189817	JN189969	[28]
550.2	2005	*Epinephelus spp.*	wild	Greece	JN189823	JN189975	[28]
6.3	2007	*D. labrax*	farmed	Greece	JN189828	JN189980	[28]
380	2008	*D. labrax*	farmed	Spain	JN189852	JN189948	[28]
487.1	2002	*E. marginatus*	wild	Greece	JN189854	JN189941	[28]
283	2009	*D. labrax*	farmed	Rovigo, Veneto, Italy	JN189865	JN189992	[28]
391.2	2008	*Epinephelus spp.*	wild	Trapani, Sicily, Italy	JN189878	JN189957	[28]
442	2001	*E. marginatus*	wild	Messina, Sicily, Italy	JN189884	JN190027	[28]
12/050	2011	*E. costae*	wild	Gulf of Annaba, Algeria	JX194165	JX194164	[31]
496.1	2011	*E. costae*	wild	Apulia, Italy	JX290519	JX290531	[16]
385.1	2011	*E. marginatus*	wild	Apulia, Italy	JX290520	JX290532	[16]
396.3	2011	*E. costae*	wild	Apulia, Italy	JX290521	JX290533	[16]
424.1	2011	*D. labrax*	wild	Apulia, Italy	JX290522	JX290534	[16]
425.2	2011	*E. costae*	wild	Apulia, Italy	JX290523	JX290535	[16]
2.5	2009	*D. labrax*	Farmed	Apulia, Italy	JX290524	JX290537	[16]
2.6	2009	*D. labrax*	Farmed	Apulia, Italy	JX290525	JX290539	[16]
469.1	2010	*D. labrax*	Farmed	Apulia, Italy	JX290529	JX290541	[16]
469.2	2010	*D. labrax*	Farmed	Apulia, Italy	JX290530	JX290542	[16]
T2.12	2012	*E. costae*	wild	Tabarka, Tunisia	KF748949	KF748941	[31]
T3.12	2012	*E. marginatus*	wild	Tabarka, Tunisia	KF748950	KF748942	[31]
T4.12	2012	*E. costae*	wild	Tabarka, Tunisia	KF748951	KF748943	[31]

B1.12	2012	*E. costae*	wild	Bizerte, Tunisia	KF748952	KF748944	[31]
T1-12	2012	*E. marginatus*	wild	Tabarka, Tunisia	KF748953	KF748945	[31]
B2.12	2012	*E. marginatus*	wild	Bizente, Tunisia	KF748954	KF748946	[31]
K1.12	2012	*E. marginatus*	wild	Kelibia, Tunisia	KF748955	KF748947	[31]
S16.12	2012	*D. labrax*	farmed	Sahel region, Tunisia	KF748956	KF748948	[31]
1	2014	*E. alexandrinus*	na	Italy	KJ939350	KJ939351	Amoroso et al., 2014_unpublished
Cabrera	2011	*E. marginatus*	wild	Cabrera, Balearic Archipelago, Spain	LC222623	LC222625	[32]
El Toro	2011	*E. marginatus*	wild	El Toro, Balearic Archipelago, Spain	LC222624	LC222626	[32]
VNNV/E.costae/Algeria/978_2/Oct19	2019	*E. costae*	wild	Collo, Algeria	MT214097	MT214099	[33]
VNNV/E.marginatus/Algeria/979_2/Sep17	2017	*E. marginatus*	wild	Oran, Algeria	MT214098	MT214100	[33]
523/2018	2018	*D. labrax*	farmed	Liguria, Italy	OP069723	OP069742	[59]
537/2018	2018	*Sparus aurata*	farmed	Lazio, Italy	OP069725	OP069745	[59]
766/20	2020	*Tritia mutabilis*	wild	Emilia Romagna, Italy	OP234318	OP251136	[60]
MmNNV1_2023	2023	*E. marginatus*	wild	Columbretes Islands, Spain	PP814714	PP814716	[34]
MmNNV2_2023	2023	*E. marginatus*	wild	Columbretes Islands, Spain	PP814715	PP814717	[34]
IZSM 101223	2024	*E. aeneus*	na	Calabria, Italy	PQ871106	PQ871114	Rinaldi et al., unpublished
IZSM 103888	2024	*D. labrax*	na	Campania, Italy	PQ871107	PQ871115	Rinaldi et al., unpublished
IZSM 93628_8	2024	*B. capriscus*	wild	Italy	PQ871108	PQ871116	[43]
IZSM 93628_10	2024	*D. volitans*	wild	Calabria, Italy	PQ871109	PQ871117	[43]
IZSM 99469	2024	*D. volitans*	wild	Italy	PQ871110	PQ871118	[43]
IZSM 99656	2024	*E. marginatus*	na	Calabria, Italy	PQ871111	PQ871119	Rinaldi et al., unpublished
IZSM 93628_9	2024	*B. capriscus*	wild	Calabria, Italy	PQ871112	PQ871120	[43]
IZSM_93628_4	2024	*E. aeneus*	na	Calabria, Italy	PQ871113	PQ871121	Rinaldi et al., unpublished
